# Schatzki's Ring in Angelman Syndrome: A Diagnostic Dilemma in Neurodevelopmentally Disabled Patients

**DOI:** 10.4021/jocmr2009.04.1234

**Published:** 2009-04-24

**Authors:** Young Sammy Choi, David Scott Sachar

**Affiliations:** a Departments of Medicine, Pediatrics, and Research, Womack Army Medical Center, Fort Bragg, North Carolina 28310, USA; b Division of Gastroenterology, Department of Medicine, Womack Army Medical Center, Fort Bragg, North Carolina 28310, USA

## Abstract

Angelman Syndrome is a neurodevelopmental condition with a characteristic phenotype that includes epilepsy and lack of communication. We describe its first reported association with Schatzki's ring that presented as a life-long history of intermittent retching. Because of associated cognitive dysfunction, careful diagnostic consideration is required to detect this underlying condition.

**Keywords:**

Schatzki's ring; Angelman Syndrome; Esophageal "B" ring; Barium esophagogram; Neurodevelopmental delay; Retching

## Introduction

Angelman Syndrome (AS) was first described in 1965 as "puppet children" characterized by severe neurodevelopmental disability, inability to speak, abnormal motor movement, easily provoked laughter, and epilepsy [1]. With the description of the genetic basis for this condition in 1987 [2], a more accurate diagnosis of this syndrome was possible. This led to the publication of the salient clinical features in a consensus statement in 1995 [3]. Ten years later, an updated consensus statement was published [4]. Other than constipation and early childhood feeding difficulties, gastrointestinal tract symptoms are not part of the phenotypic features of AS. We present a case of a 26-year-old woman with AS and a life-long history of intermittent retching.

## Case Report

A 26-year-old female with AS and documented maternal deletion of chromosome 15(q11-13) presented with 1 month history of worsening retching, 3.6 kg weight loss, decreased oral intake, and depressed affect. Medications were temazepam, valproate, diazepam, liothyronine, and omeprazole taken on a scheduled basis.

Eighteen months prior she developed protracted retching associated with a 2.3 kg weight loss during which time an upper gastrointestinal contrast study (UGI) was unremarkable and esophagogastroduodenoscopy (EGD) showed only mild chronic gastritis. She was treated with proton-pump inhibition and recovered over the next few weeks. As a child, she had similar retching and after negative gastrointestinal evaluation, her symptoms were somewhat relieved with propranolol in an attempt to treat possible abdominal migraines. Electroencephalographic monitoring during symptoms remained at baseline. With time, she continued to improve and propranolol was discontinued though she still had inexplicable short-lived episodes of retching. Two weeks before presentation, a contrast esophagram (CE) was normal.

Examination at presentation was remarkable for a weight of 45.4 kg and a body mass index of 18.0. Findings from baseline included depressed affect and a 3.0 x 2.5 cm mons pubis abscess. Complete blood count, comprehensive metabolic panel, and thyroid function studies were normal.

The patient was admitted for drainage of her abscess under general anesthesia during which time repeat EGD was performed. A Schatzki's ring (SR) with a residual lumen of 10 mm was diagnosed and subsequently dilated to 20 mm; no additional findings were discovered. Her proton-pump inhibition was continued. Immediately after recovering from her procedure, retching subsided, appetite increased, and her typical happy demeanor returned.

## Discussion

This is the first reported case of SR occurring in association with AS. SR or esophageal "B" ring is a thin circumferential fold of mucosa found in the distal esophagus [5]. Most patients with symptomatic SR present after 40 years of age and its etiology remains debatable [6]. Patients with a esophageal luminal diameter of < 13 mm will be symptomatic while those with a lumen in excess of 20 mm will rarely be so; those between 13 and 20 will vary [7]. Other than constipation and early childhood feeding problems mentioned in the Consensus Conference statements [3, 4], gastroesophageal reflux secondary to obesity has been the only other gastrointestinal finding reported with AS [8]. Of note, our patient's prior episodes of retching (also previously unreported) remain an enigma.

We feel our described association between AS and SR is a chance occurrence whose significance is not necessarily in being the first reported case, but in that it is descriptive of the diagnostic dilemmas found in AS or similarly developmentally delayed patients. Confounding all AS cases is the severe compromise in communicability and therefore the most important part of the diagnostic process, history, is lacking. SR typically presents with progressive solid phase dysphagia [6]. Our patient presented acutely with worsening retching, decreased oral intake, and weight loss with a background history of intermittent retching and nearly identical symptoms 18 months earlier. All prior episodes slowly improved without determination of a clear etiology.

CE or many times an UGI in children is considered the diagnostic procedure of choice. However a high index of suspicion is necessary in order to alert the radiology team so that proper full-column prone technique [6, 9-11] can be performed accompanied by respiratory maneuvers such as Valsalva [10]. EGD is less sensitive particularly when the luminal diameter is not as severely restrictive [9, 10] and it may be particularly ineffective in children [12]. Furthermore, whether CE or EGD is performed, the diagnosis can be difficult to make even in patients with typical presentations and no developmental problems; delays in diagnosis have been reported to average 6 years [13]. In our patient, routine CE was normal just 2 weeks before the etiology was discovered.

AS and other severely developmentally delayed patients may present in ways that appear unusual simply because they cannot communicate their symptoms. In our patient, CE was an innocuous procedure to perform. However, we were not suspicious for a SR so specific technique and positioning was not made. Additionally, respiratory maneuvers such as Valsalva are not possible to perform in patients with significant developmental disabilities as found in patients with AS. Though we were fortunate EGD was diagnostic (endoscopy may not be sensitive until a tight stricture is present), the decision to pursue this study is not as automatic as it would be in other patients. This is because many AS patients are considered American Society of Anesthesiology physical status Class III [14]. Therefore, EGD would require coordination with anesthesiology [15] and consideration of general anesthesia, something that is not without risk in AS patients [16-18]. Such endoscopic procedures for this type of patient require careful consideration rather than a "shot-gun" approach.

It was rather fortuitous that this patient presented with an abscess that necessitated operative treatment under general anesthesia. This afforded an opportune time to perform EGD with little added risk. Had this patient not required anesthesia for another indication, EGD might not have been performed as quickly due to her previous evaluations for similar presentations. As this case illustrates, upper gastrointestinal tract symptoms in patients with severe developmental conditions such as AS require a thoughtful approach to diagnosis.
